# Physician and Surgeon Communication Assessed via the Pathology Requisition in a Regional Laboratory Over Ten Years

**DOI:** 10.7759/cureus.27714

**Published:** 2022-08-05

**Authors:** Michael Bonert, Uzma Zafar, Phillip Williams, Ihab El-Shinnawy, Rosalyn A Juergens, Asghar Naqvi, Jean-Claude Cutz, Christian Finley, Pierre Major, Anil Kapoor

**Affiliations:** 1 Pathology and Molecular Medicine, Division of Anatomical Pathology, McMaster University, Hamilton, CAN; 2 Pathology, Monmouth Medical Center, Long Branch, USA; 3 Pathology and Laboratory Medicine, St. Barnabas Medical Center, Livingston, USA; 4 Oncology, Division of Medical Oncology, McMaster University, Hamilton, CAN; 5 Surgery, Division of Thoracic Surgery, McMaster University, Hamilton, CAN; 6 Surgery, Division of Urology, McMaster University, Hamilton, CAN

**Keywords:** pathology, effective communication, physician-physician communication, patient handover, consultation, clinical history, communication with pathology

## Abstract

Background

Ineffective communication between healthcare providers is a known risk factor for adverse events.

Objective

The aim of this study was to retrospectively assess the communication with pathology via an analysis of the information provided on the pathology requisitions over ten years.

Methods

All in-house surgical specimens and all non-gynecologic cytopathology specimens accessioned from 2011 to 2020 were retrieved at a regional laboratory. Cases with any clinical information were deemed to have a clinical history present (CHP). CHP was tabulated by submitting physicians/surgeons (SPS), hospital site, year, and tissue group.

Results

The study period contained 554,817 relevant pathology reports, of which 553,966 could be extracted. The overall CHP rate was 74% and varied from 76% to 67% over the study period. SPSes submitting ≥200 cases (n=314) had a mean/median/standard deviation/max/min CHP rate of 81%/92%/23%/100%/5%. The CHP varied between four hospital sites, from 53% to 97%. CHP varied from 61% to 99% by tissue group.

Conclusions

CHP is associated with several factors and appears to depend on the hospital culture, specialty, and individual physician/surgeon. The pathology requisition is a way to measure and track the communication that is clinically relevant. Improving communication with pathologists/the pathology department will likely require process changes and mandates. Hospital and laboratory accreditation bodies should consider effective communication with pathology a marker of quality and an accreditation issue.

## Introduction

A large mature body of evidence demonstrates that effective communication is a key factor in managing complex environments, such as aviation and space travel [1-3]. Thus, it is not surprising that communication failures between healthcare providers (in complex care environments) are a well-documented cause of adverse events [4]. Accordingly, communication with pathology is also recognized as important and considered a quality metric [5].

A large multi-institution study demonstrated that pathologists need information from the clinic to arrive at an accurate diagnosis [6]. In the legal context, submitting physicians and surgeons may be required to provide a relevant history (as in Ontario [7]), and it may even be specified what information should be supplied within a consultation request (as in Poland [8]).

A local perspective and assessment

A prior local project examined clinical history provision in the context of prostate core biopsies and found significant variation among submitting physicians/surgeons [9]. An earlier version of this work was presented in poster form at the European Congress of Pathology in 2019 in Nice, France. There appears to be a significant difference between actual practice and the known fact that suboptimal communication is associated with suboptimal care.

Objective of study

The goal of this study is to objectively assess communication with pathology in several hospitals served by a regional pathology laboratory over several years. Additional goals are to investigate whether hospital site, department (as assessed by the surrogate of ‘tissue group’), and individual physician/surgeon are predictors of clinical history present.

## Materials and methods

Approval from the Hamilton Integrated Research Ethics Board (HiREB; approval no. 4879) was obtained to extract all pathology reports over a period of ten years and examine the provided clinical history in relation to the healthcare providers. The data was fully anonymized with respect to patient identifiers and healthcare providers. Consent was not obtained as the data were completely anonymized with regard to patient identifiers and healthcare providers.

The anonymized data was fed into a custom program written in Python (https://www.python.org/) that classified cases as clinical history present (CHP) and categorized them into one or more tissue groups, based on a dictionary that was created for that task (Appendix A). A further custom program in Python outputs the anonymized data in a coded format readable by R (https://cran.r-project.org; RStudio, Boston, MA). Subsequently, the tabulations and plots were made with R.

The clinical history present (CHP) status was determined by searching for in-house standardized phrases ('Not available on requisition' for surgical cases and 'Clinical history not provided' for cytology cases) and (if applicable) examining the length of clinical history. If one of the standardized phrases was found the case was marked as ‘NAOR’ (not available on requisition). As some reports did not have a clinical history section at all: cases that were not ‘NAOR’ were further examined to determine the length of the clinical history section (number of characters). All cases where the clinical history had a zero-character length were labelled ‘no information available’ (NIA). All cases that were ‘NAOR’ or ‘NIA’ were labelled ‘incomplete’. All cases not ‘incomplete’ were deemed clinical history present (CHP).

The tissue group (TG) was meant to be a rough surrogate for the originating (clinical) department; thus, each case was only assigned to one TG in the post-processing in R. In this context, cases were arranged in a hierarchy (Appendix B). ‘Cytology’ was set very high in the hierarchy to separate these from the surgical cases.

Case complexity was captured using the number of tissue blocks and the workload units (L4E 2018), as described in a prior study [10]. Each specimen was categorized into one of four mutually exclusive categories (‘biopsy’, ‘resection’, ‘ambiguous’, ‘unknown’) using a string-matching algorithm.

The regional laboratory (Hamilton Regional Laboratory Medicine Program) consists of multiple hospital sites. Pathology cases were identified by the hospital site in which the specimen was obtained/accessioned [St. Joseph’s Healthcare Hamilton (SJHH), Juravinski Hospital (JH), McMaster University Medical Centre (MUMC), or Hamilton General Hospital (HGH)]. The clinical departments are under the umbrella of two hospital organizations: SJHH and Hamilton Health Sciences (JH, MUMC, HGH).

Submitting physicians/surgeons were assigned to a hospital site on the basis of the hospital site where the largest number of specimens they submitted came from/were accessioned. It should be noted that the term ‘submitting physician/surgeon’ in this study corresponds to the field 'requesting physician' on the pathology report; invariably, this field on the pathology report is synonymous with the term 'most responsible physician [or surgeon]'. The hospital sites were anonymized in the results.

## Results

The pathology reports were successfully extracted for the study period; however, it was noted that the default report format did not contain any history for gynecologic cytopathology cases. This discovery led to a change in the process such that the clinical history is now provided; however, the data were not re-extracted for the purpose of this study. Thus, the gynecologic cytopathology cases were excluded from the analysis.

The study period (2011-2020), after excluding gynecologic cytopathology cases, contained 554,817 relevant pathology reports, and 553,996 of these could be extracted. The no information available (NIA) cases (cases without a ‘clinical history’ section) numbered as high as 3,815 per year. To draw attention to the absent clinical history, a change within the pathology department was instituted in 2017; the phrases 'not available on requisition' (surgical cases) and 'clinical history not provided' (cytology cases) were used as a space holder if no history was provided. The ‘NIA’ cases subsequently decreased to 44 out of 46,652 in the year 2020 (trend in Appendix-Supplemental Figures).

**Figure 1:**
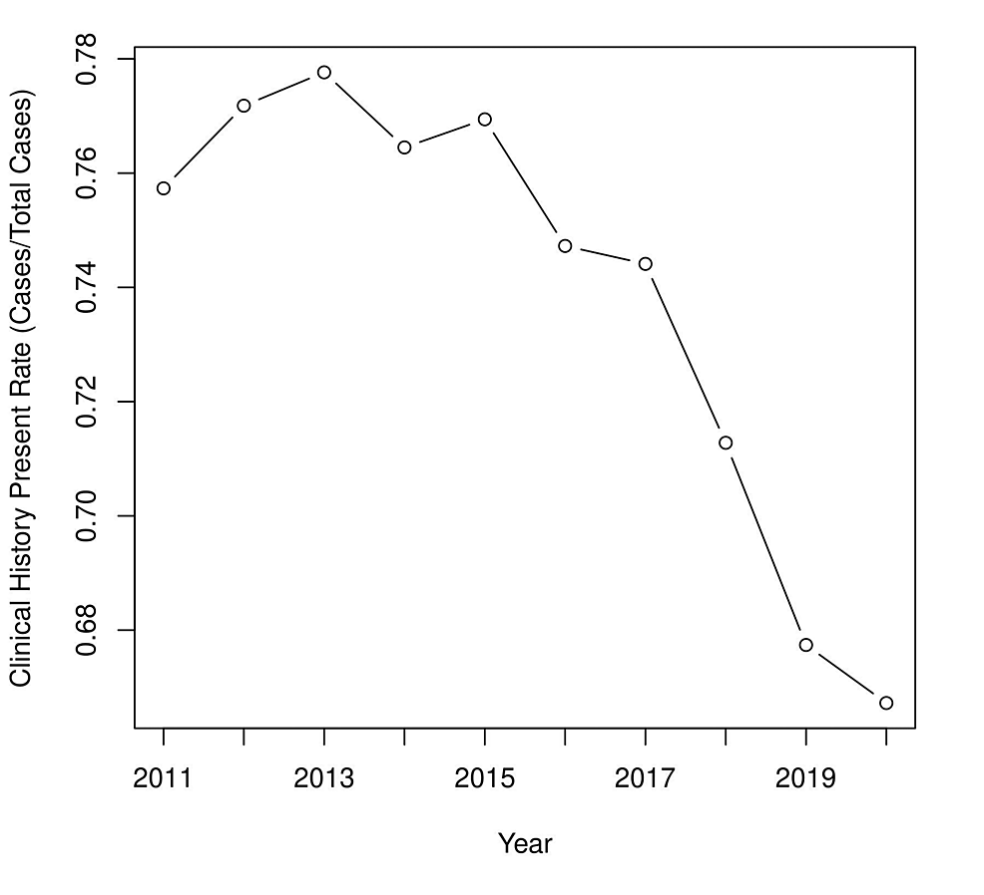
Overall clinical history present rate by year

In the study group, 553,960 reports could be classified. Over the study period, 410,147 cases (74%) were CHP. CHP varied significantly by year (Figure 1) and decreased at the end of the study period.

**Figure 2:**
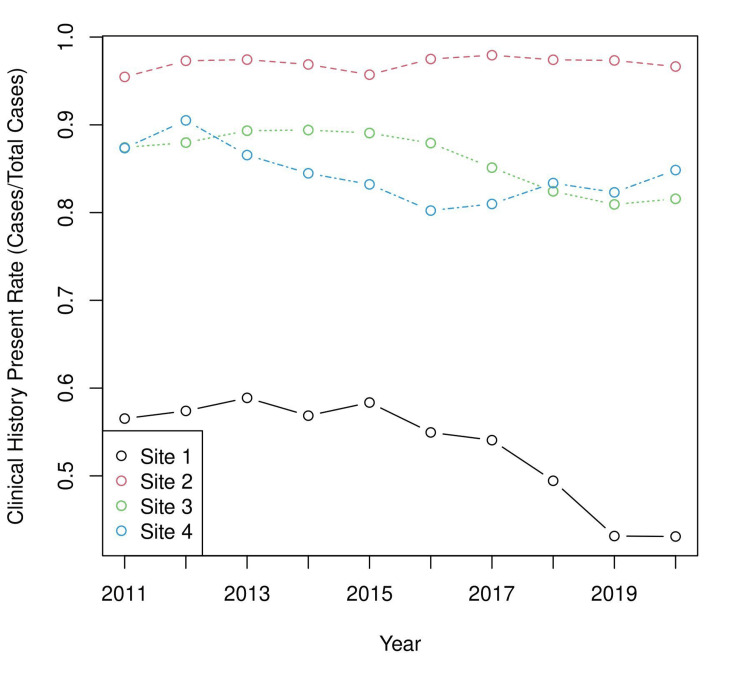
Clinical history present rate by site and year

The hospital sites 1, 2, 3, and 4 had 242,563, 123,041, 137,720, and 50,636 cases over the study period, respectively. A sub-analysis by hospital site (Figure 2) showed that significant differences existed between the different hospital sites and that there was a significant decrease in the overall CHP rate. The data presented in Figure 2 suggest that the overall CHP rate decrease can be attributed primarily to changes at ‘Site 1’.

**Figure 3:**
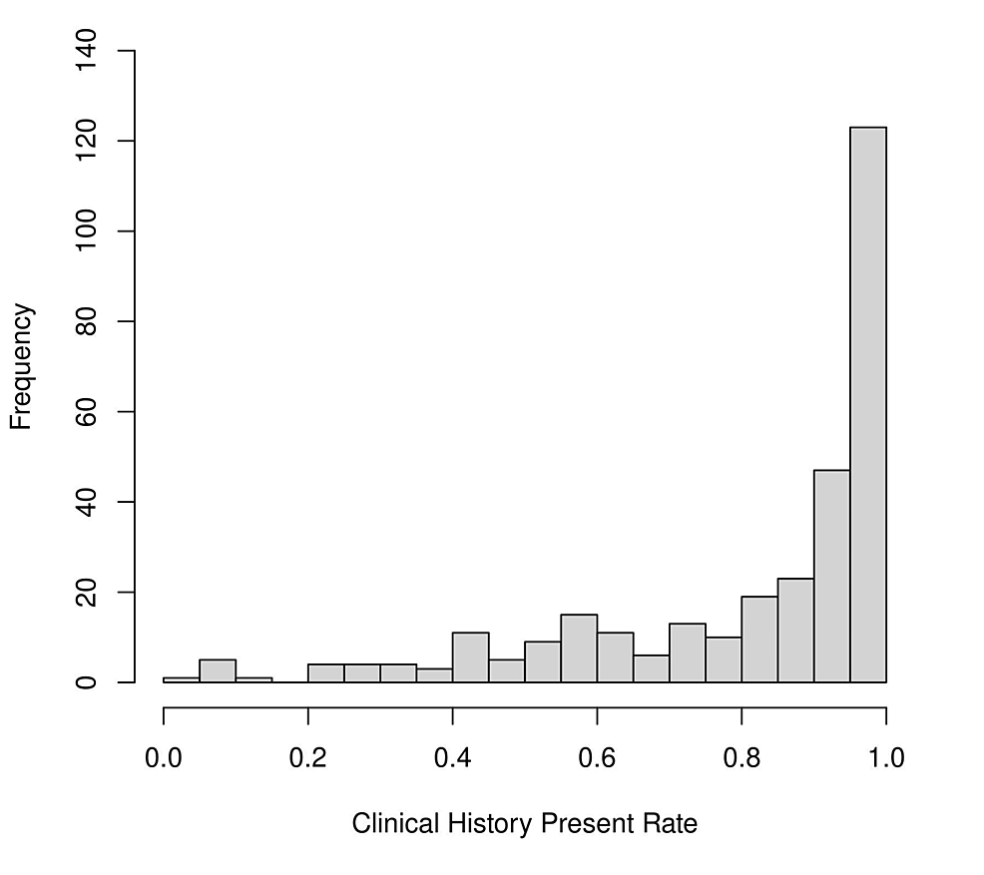
Histogram of clinical history present rate for all sites for MDs submitting ≥200 cases

There were 314 SPS that submitted at least 200 specimens each. Among the 314, the mean/median/standard deviation/maximum/minimum number of specimens were 1609.0/837.5/1827.4/9,913/203. The effect of the individual SPS was examined in Figure 3; it shows significant variation between individuals submitting physicians/surgeons. A kernel density plot for SPS versus completeness is in the Appendix-Supplemental Figures. A weak negative association is seen between volume submitted and CHP; high volume submitters tended to have lower CHP rates (Appendix-Supplemental Figures).

The 314 submitting physicians/surgeons by hospital site were 118, 85, 74, and 37. Histograms and kernel density plots for the individual sites show significant variation (Appendix-Supplemental Figures). The CHP rates varied significantly by the tissue group and are shown in Table 1. The specimens were categorized by specimen intent (biopsy 299,203; excision 170,864; ambiguous 42,105; unknown 41,788). Completeness by intent for biopsy, excision, ambiguous, and unknown was 69%, 77%, 84%, and 86%, respectively. CHP in logistic regression was individually predicted by case complexity (L4E2018 pathologist workload units, number of tissue blocks), specimen intent, tissue group, year, and hospital site (all p<0.0001).

**Table 1:** Volume and clinical history present by (mutually exclusive) tissue group CHP: clinical history present, CVS: cardiovascular system, Unknown: case could not be classified but ‘source of specimen’ retrieved, Unclassified: ‘source of specimen’ not found (n=35) or unclassified in categories above (e.g., 'c_mass').

Tissue group	Volume	CHP	CHP rate	Fraction of total cases
Gastrointestinal	185,863	113,483	0.611	0.336
Cytology	111,305	74,589	0.670	0.201
Gynecologic	64,413	58,974	0.916	0.116
Skin	39,006	32,436	0.832	0.070
Urology	35,958	28,110	0.782	0.065
Breast	22,877	20,703	0.905	0.041
Head and neck	16,280	12,948	0.795	0.029
Miscellaneous	15,003	12,405	0.827	0.027
Pulmonary	13,292	10,093	0.759	0.024
Soft tissue	10,590	10,350	0.977	0.019
Placenta	9222	9164	0.994	0.017
Neurologic	8679	8409	0.969	0.016
CVS	7970	7094	0.890	0.014
Lymph node	3857	3534	0.916	0.007
Unknown	3720	3195	0.859	0.007
Endocrine	3664	2653	0.724	0.007
Unclassified	1403	1219	0.869	0.003
Fetus	447	418	0.935	0.001
Hematologic	411	370	0.900	0.001
Whole cohort	553,960	410,147	0.740	1.000

## Discussion

The provision of clinical history can be assessed via the pathology requisition and it is apparent that it varies due to multiple factors. The individual submitting physician/surgeon, hospital site, and tissue type are all strong predictors. It should be noted that the SPS and hospital sites are not independent, as most SPS predominantly work at one of the institutions.

A limitation was that the threshold for CHP was very low in this study. Further analysis work could be done on the basis of the clinical history length. A partial analysis was done on the content of the clinical history using a large dictionary of terms. This analysis was put aside, as the approach was computationally expensive. More work remains on assessing the quality of the provided information within the context of the case, in order to assess whether it is relevant, important, and correct, based on what should be known to optimally manage the case. A further limitation is that the tissue group is a crude approximation of the department. A more complex analysis might attempt to (1) classify cases by string-matching, (2) assign each SPS to a tissue group, (3) re-construct the departments from the SPS’ primary tissue group, and (4) calculate the CHP by the department from the sum of the SPS assigned to the tissue group. The individual that added the clinical history information (if present) cannot be ascertained in this study; the information was not recorded on the pathology report. This also represents a limitation in the analysis. It is possible that the communication between the submitting physician/surgeon and their delegate(s) is suboptimal. Further analysis on the communication with the submitting physician/surgeon and their delegate(s), using other information sources, may be informative.

The CHP has decreased with time, despite some efforts to raise awareness about the issue and the issue rising to the level of the hospital’s central quality committee ('Medical Advisory Committee'). The time period also encompassed a major transition in 'Site 1’s' hospital’s electronic patient records. In the context of the transition, the proposal to make the provision of clinical history mandatory was not adopted. This was done despite a process change that would potentially change the hospital’s liability: all health professionals (e.g., nurses) delegated to submit a pathology case would henceforth be documented in the patient record, thus better establishing the pathology case chain of custody.

It is said that there are three big questions in pathology: (1) What is it (biopsies)? (2) Did I get it all (resections)? (3) Did I get the right thing (everything else)? The lower CHP rate in biopsies (69%) compared to excisions (77%) is a concern; when the clinical history is most likely to be needed, it is more commonly absent.

Lack of a relevant clinical history can make the triage of cases difficult in pathology. In busy practice environments with a minimal reserve service capacity, it is more likely that in busy times, urgent cases are inappropriately triaged and may be delayed. Lack of a relevant clinical history can affect how specimens are handled within the laboratory. For example, if the clinical history is a 'mass lesion', deeper cuts are prepared immediately. In liver biopsies, if the indication is suspected medical liver disease, special stains are done.

The CHP rate found (74%) is low in relation to a prior large-scale multi-institution study that included 1,004,115 specimens; the prior study found that the clinical history was missing in 2.4% of cases [11].

Input and output mismatch

Pathologists (via organizations like the College of American Pathologists) have worked on creating a workflow conducive to higher quality and more complete reports [12]. Pathologists have done much on the 'output' side of the lab; these have increased completeness [13] and standardized communication [14]. Communication from pathology, in relation to reporting, is regulated (in California) to allow the systematic rapid analysis of health data [15,16].

Improvements on the 'input' side of the laboratory are overdue and ideally should be standardized into a structured form with elements relevant to the particular submitted specimen, so it mirrors the 'output' side of the laboratory, that is, increasingly synoptic type pathology reports.

Objective continued measurement

The computerization of medical records presents ongoing challenges and opportunities in communication with pathology [17]. Measuring communication is becoming easier with the further computerization of medical records. Adding more measurements and refining them will be essential for improving communication. In the absence of continuous measurements and the assessment of communication, there can be no evidence-based dialogue about how to improve communication and what is effective to bring about sustained improvement.

How the crude measurement of clinical history completeness herein is related to hard outcomes (number of medical legal actions, patient harm) remains to be established. Prior work does show that a deficient clinical history leads to delayed reporting and increased work in pathology [6].

Possible causes

Clinical History is an Externality

An externality is a cost (or benefit) that is borne by a third party. In our practise environment, submitting physicians/surgeons are compensated by a single (government) payer for the services rendered. Pathologists in this context are third parties, and the clinical history is an externality; the presence or absence of a provided clinical history does not affect the SPS compensation.

Since providing a clinical history requires time, there is a significant disincentive to doing so. Costs associated with a deficient history are primarily borne by the pathologist. An adverse event that is associated with a deficient history is, primarily, a cost to the patient. Additional costs may be incurred by physicians/surgeons that treat the sequelae of an adverse event.

Indirectly, all medico-legally insured physicians/surgeons pay for deficient histories that lead to medico-legal insurance payouts. Thus, submitting physicians/surgeons who judiciously and consistently supply a clinical history are paying for individuals who do not-through higher medico-legal insurance rates.

Power Dynamic and Standards

The state of communication with pathology is likely a reflection of referral patterns and the power dynamic in physician/surgeon and pathologist interactions. Submitting physicians/surgeons often decide where to send the specimens they generate. Thus, pathologists (and pathology practices) may face repercussions when asking for a complete relevant history. In the context of competition between pathology practices, the provision of clinical history may be a casualty of catering to the customer. Accreditation standards that require a clinical history (with audits to ensure the provided information from the submitting physician/surgeon is relevant and sufficient) would break a dynamic that likely leads to a lower standard of care with adverse effects for patients.

Ideally, submission of a specimen to pathology without a clinical history should not be possible (in the context of electronic physician/surgeon pathology order entry), as is the case in one of the hospital sites for requested radiology tests.

## Conclusions

The provision of clinical history can be measured from the pathology requisition. Whether information is provided can be predicted by the hospital site, individual submitting physician/surgeon, and type of specimen.

Improving communication with pathology will likely require process changes and mandates. Hospital and laboratory accreditation bodies should consider communication with pathology a quality metric and make accreditation contingent on a minimum standard of communication from submitting physicians/surgeons.

## Figures and Tables

**Figure 4:**
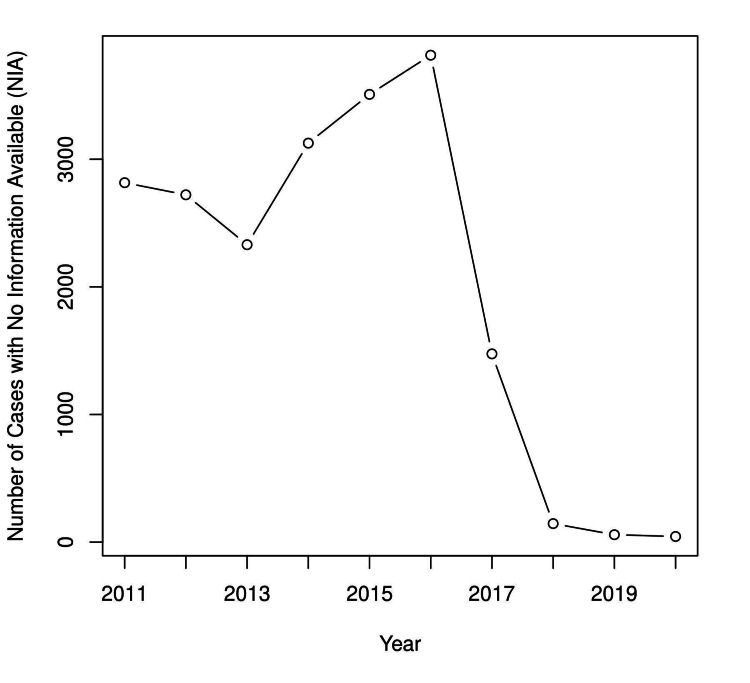
Cases with no information available by year

**Figure 5:**
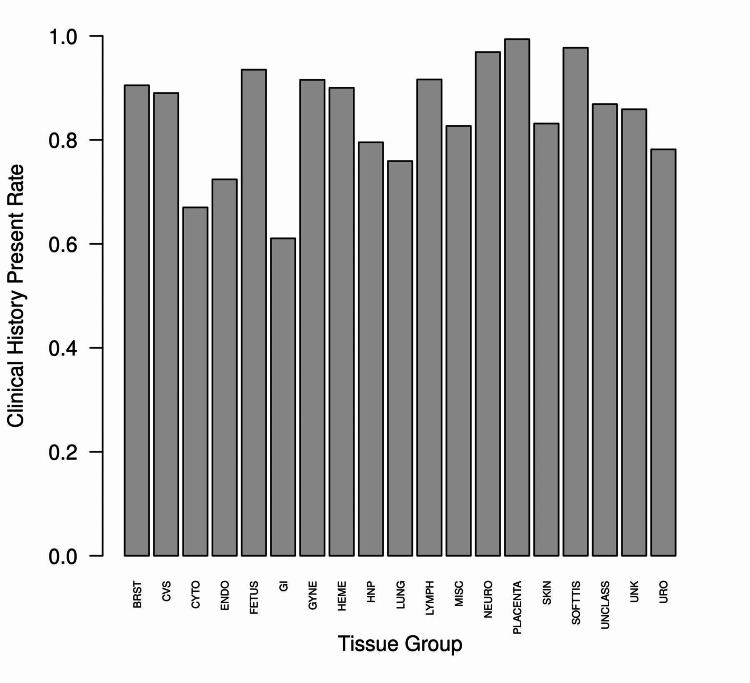
Clinical history present by tissue group Shows data in Table 1

**Figure 6:**
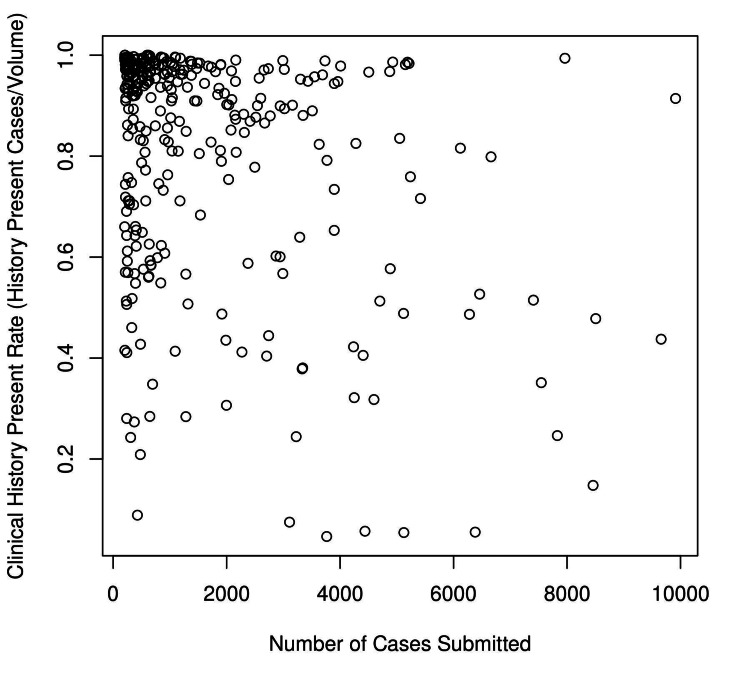
Clinical history present by submitting MD and volume submitted Each circle or 'o' represents one or more submitting MDs.

**Figure 7:**
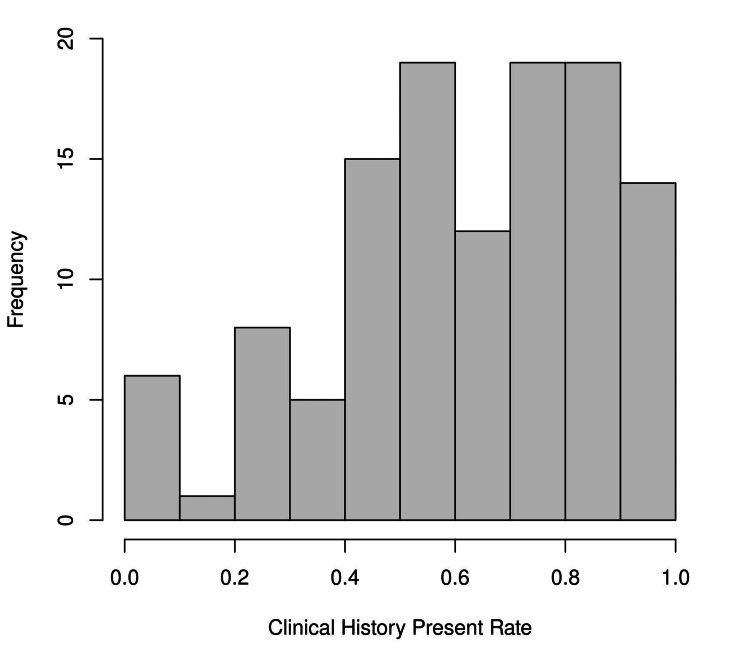
Histogram of clinical history present - site 1

**Figure 8:**
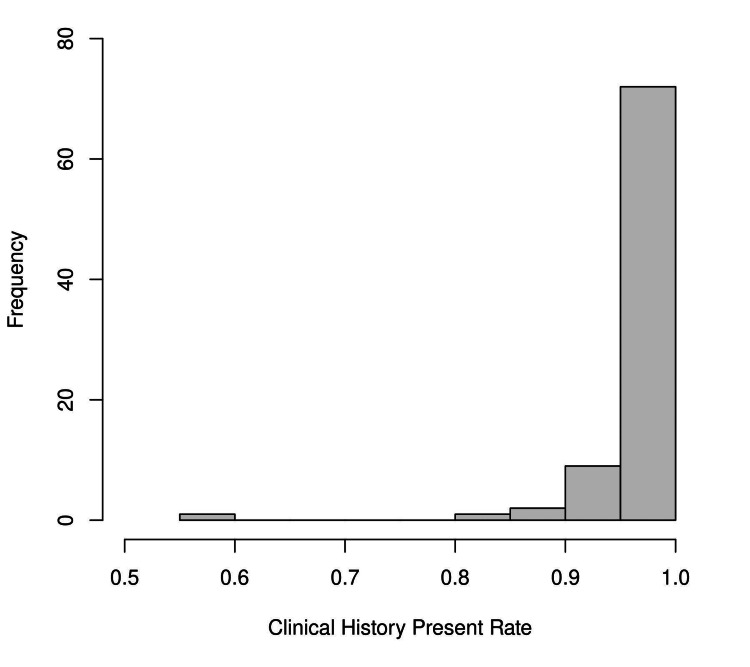
Histogram of clinical history present - site 2

**Figure 9:**
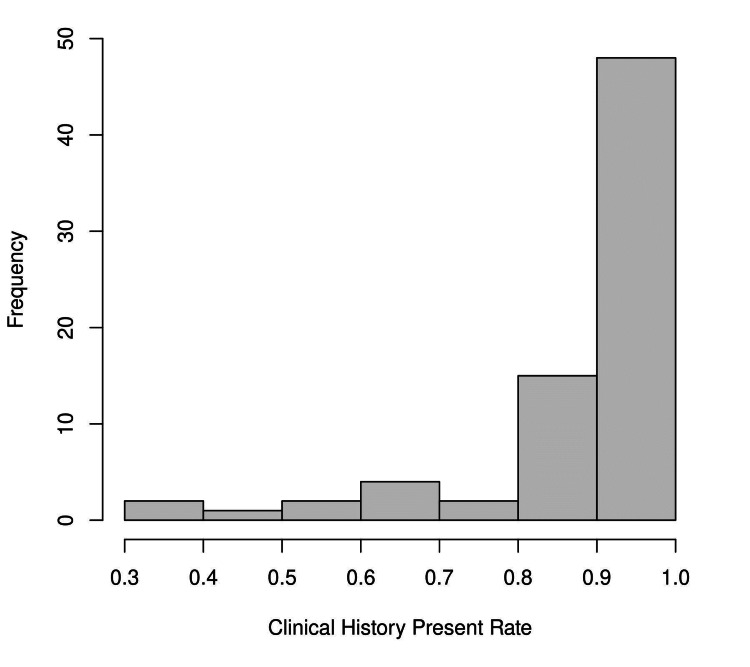
Histogram of clinical history present - site 3

**Figure 10:**
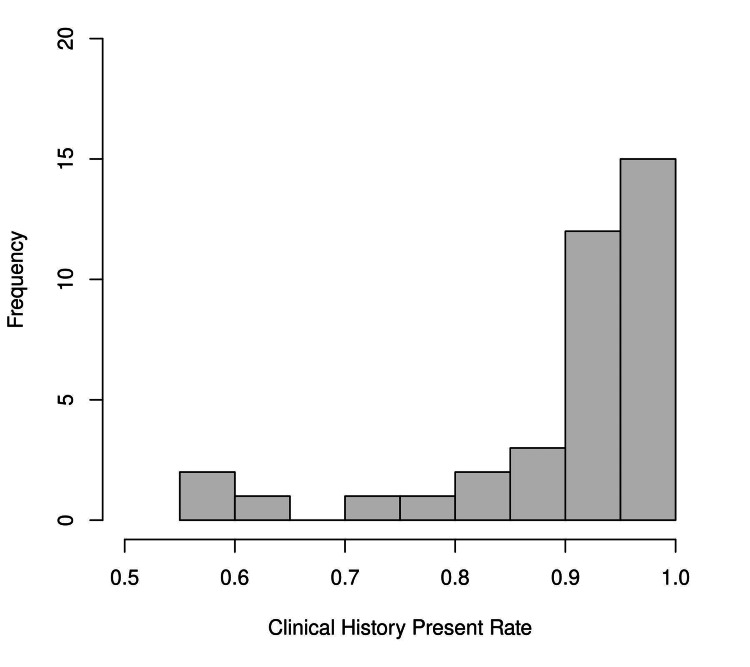
Histogram of clinical history present - site 4

**Figure 11:**
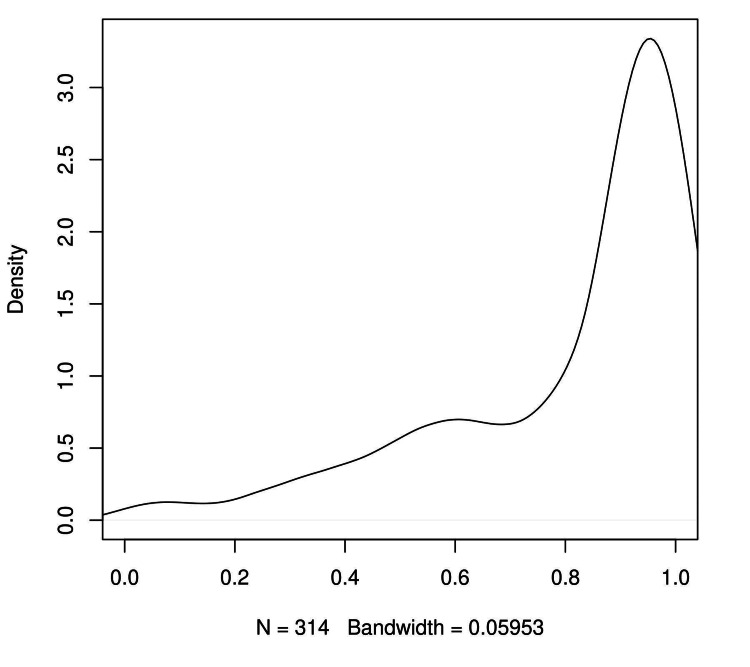
Kernel density plot of clinical history present – all sites

**Figure 12:**
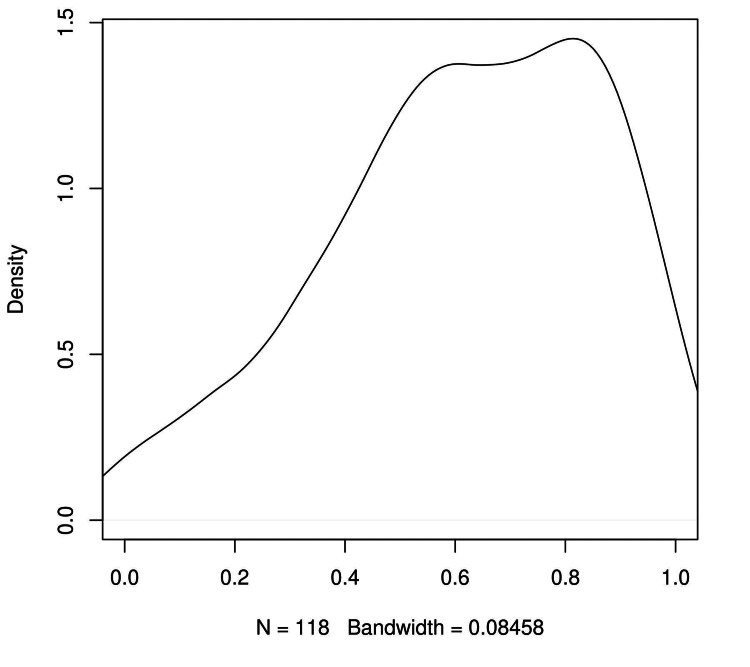
Kernel density plot of clinical history present - site 1

**Figure 13:**
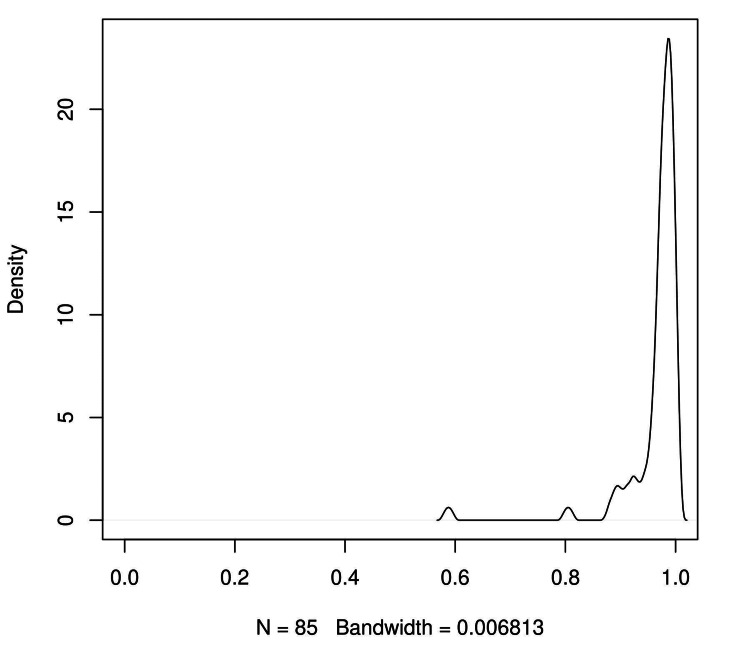
Kernel density plot of clinical history present - site 2

**Figure 14:**
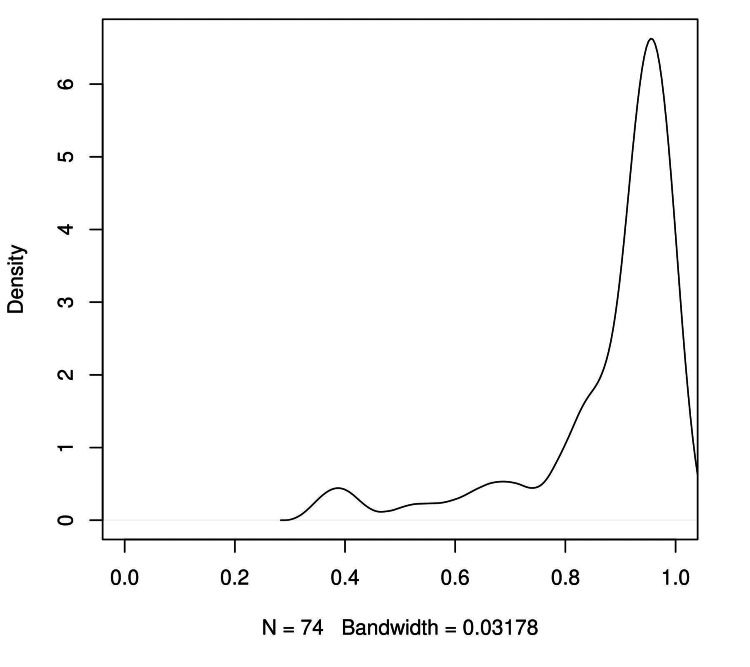
Kernel density plot of clinical history present - site 3

**Figure 15:**
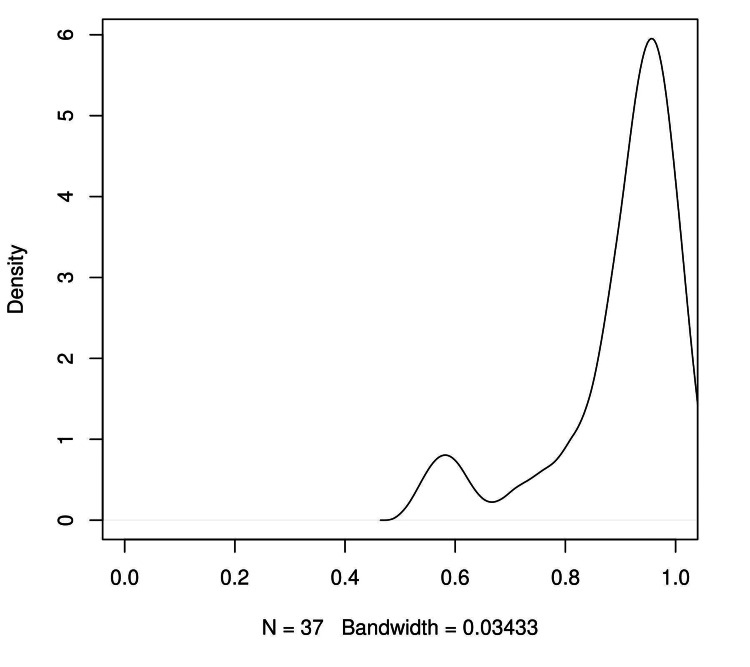
Kernel density plot of clinical history present - site 4

## Appendices

Appendix A

**Table 2:** Category and Text

Category	Term
c_cyto	FNA
c_cyto	FINE NEEDLE ASPIRATION
c_cyto	BAL
c_cyto	URINE
c_cyto	CEREBROSPINAL
c_cyto	PERITONEAL WASH
c_cyto	FLUID FROM RIGHT OVARIAN CYST
c_cyto	FLUID FROM LEFT OVARIAN CYST
c_cyto	PERITONEAL FLUID
c_cyto	PLEURAL FLUID
c_cyto	ESOPHAGEAL BRUSH
c_cyto	PAP SMEAR
c_cyto	anal swab
c_cyto	BRONCHIAL ALVEOLAR LAVAGE
c_cyto	BRONCHIAL BRUSH
c_cyto	BRONCHIAL WASH
c_cyto	PERICARDIAL FLUID
c_cyto	BRONCHIAL ASPIRATE
c_cyto	SPUTUM
c_cyto	MISCELLANEOUS CYTOLOGIC MATERIAL
c_brst	breast
c_brst	nipple
c_endo	thyroid
c_endo	adrenal
c_endo	parathyroid
c_gi	colon
c_gi	sigmoid
c_gi	colectomy
c_gi	rectum
c_gi	anus
c_gi	anal
c_gi	cecum
c_gi	esophagus
c_gi	GEJ
c_gi	Gastro-esophageal
c_gi	stomach
c_gi	gastric
c_gi	duodenum
c_gi	small bowel
c_gi	small intestine
c_gi	bowel
c_gi	ileum
c_gi	pancreas
c_gi	jejunum
c_gi	appendix
c_gi	gallbladder
c_gi	gall bladder
c_gi	liver
c_gi	ampulla
c_gi	Ileocecal valve
c_gi	Anal Tissue
c_gi	Hemorrhoidal Tissue
c_gi	bile duct
c_gi	pylorus
c_gi	Ileocecal
c_gyne	ovary
c_gyne	uterus
c_gyne	cervix
c_gyne	Fallopian
c_gyne	uterine tube
c_gyne	endometrium
c_gyne	vagina
c_gyne	vulva
c_gyne	ciltorus
c_gyne	labia
c_gyne	labium
c_gyne	Bartholin
c_gyne	conception
c_hnp	larynx
c_hnp	nasal polyp
c_hnp	thyroid
c_hnp	tongue
c_hnp	mouth
c_hnp	cheek
c_hnp	pharynx
c_hnp	oral
c_hnp	GINGIVA
c_hnp	tonsil
c_hnp	Adenoids
c_hnp	cord
c_hnp	salivary
c_hnp	parotid
c_hnp	submandibular
c_hnp	epiglottis
c_hnp	Ethmoid
c_hnp	THYROHYOID
c_hnp	lip
c_hnp	uvula
c_hnp	Palate
c_hnp	Mandible
c_hnp	maxilla
c_hnp	jaw
c_hnp	Nasal turbinates
c_hnp	tooth
c_hnp	nasal
c_hnp	cholesteatoma
c_neuro	brain
c_neuro	spinal cord
c_neuro	sural nerve
c_neuro	muscle
c_neuro	skull
c_lung	lung
c_lung	lingula
c_lung	bronchus
c_lung	trachea
c_lung	carina
c_lung	pleura
c_lung	rib
c_fetus	Fetus
c_placenta	placenta
c_softtis	lipoma
c_softtis	retroperitoneal
c_softtis	retroperitoneum
c_softtis	bone
c_uro	bladder
c_uro	urinary
c_uro	kidney
c_uro	renal
c_uro	penis
c_uro	testis
c_uro	prostate
c_uro	seminal vesicle
c_uro	ureter
c_uro	urethra
c_uro	scrotum
c_uro	epididymis
c_uro	vas deferens
c_cvs	heart
c_cvs	thrombus
c_cvs	aortic valve
c_cvs	mitral valve
c_cvs	tricuspid
c_cvs	artery
c_cvs	aorta
c_skin	skin
c_skin	back
c_skin	shoulder
c_skin	neck
c_skin	face
c_skin	ear
c_skin	nose
c_skin	arm
c_skin	leg
c_skin	shin
c_skin	foot
c_skin	chest
c_skin	eyelid
c_skin	eye lid
c_skin	canthus
c_skin	scalp
c_skin	forehead
c_skin	temple
c_skin	thigh
c_medkid	kidney
c_paeds	hirschsprung
c_heme	spleen
c_heme	thymus
c_heme	bone marrow
c_heme	ISIS
c_lymph	lymph node
c_misc	hernia sac
c_misc	Dupuytren
c_misc	palmar
c_misc	pannus
c_misc	eye
c_misc	cornea
c_misc	Conjunctiva
c_misc	omentum
c_misc	intervertebral disc
c_misc	vertebra
c_misc	finger
c_misc	thumb
c_misc	hand
c_misc	wrist
c_misc	pelvis
c_misc	perineum
c_misc	nerve
c_misc	fat
c_misc	foreign body
c_misc	Intrauterine Device
c_misc	synovium
c_misc	scapula
c_misc	femur
c_misc	femoral head
c_misc	femoral capsule
c_misc	hip
c_misc	knee
c_misc	synovium
c_misc	synovial
c_misc	mediastinum
c_misc	mediastinal
c_misc	pericardium
c_misc	peritoneum
c_misc	abdominal wall
c_misc	groin
c_mass	mass
c_mass	lesion
c_mass	tumour
c_mass	tumor

Appendix B

**Table 3:** Category hierarchy The lowest in the hierarchy is UNK (1). The highest in the hierarchy is FETUS (19).

Priority	Category
1	UNK
2	MISC
3	HEME
4	LYMPH
5	SKIN
6	SOFTTIS
7	HNP
8	ENDO
9	BRST
10	GI
11	GYNE
12	LUNG
13	URO
14	CVS
15	NEURO
16	PLACENTA
17	CYTO
19	FETUS

Appendix - Supplemental Figures

The supplemental figures (Figure 4 and Figures 6-15) show additional information in the underlying data set. Figure 5 show the data in Table 1.

Data availability

The raw anonymized data set can be requested.
